# Multivariate Optimization of an Eco-Friendly Zinc-Based MOF for Adsorptive Removal of Emerging Contaminants in Food Samples

**DOI:** 10.1021/acsomega.5c05658

**Published:** 2025-09-11

**Authors:** Wesley C. P. Aquino, Iare S. Ribeiro, Marcos V. S. Pereira, Luciano M. Guimarães, Gilberto R. da Silva-Junior, Renê C. da Silva, Jemmyson R. de Jesus

**Affiliations:** † Research Laboratory in Bionanomaterials, LPbio, Department of Chemistry, Federal University of Viçosa, 36570-900 Viçosa, Minas Gerais, Brazil; ‡ Department of Physics, Federal University of Viçosa, 36570-900 Viçosa, Minas Gerais, Brazil

## Abstract

This work reports the development and application of a sustainable zinc-based coordination compound, [Zn­(Bz-COO)_2_]_∞_, for the detection of emerging contaminants, specifically the synthetic dyes alizarin violet (AV) and methylene blue (MB), in shrimp samples. The material was characterized using Fourier transform infrared spectroscopy, scanning electron microscopy, powder X-ray diffraction, thermogravimetric analysis, Brunauer–Emmett–Teller surface area analysis, and Raman spectroscopy. The extraction of contaminants was performed using multivariate optimization evaluating (i) adsorbent-to-sample ratio (1:1, 1:5, and 1:10 m/m) and (ii) eluent nature (low, medium and high polarity). As a result, the coordination compound presented a high specific surface area (5216 m^2^ g^–1^) and pore volume of 0.0136 cm^3^ g^–1^. Adsorption studies confirmed a strong affinity of [Zn­(Bz-COO)_2_]_∞_ for AV and MB, with maximum adsorption capacities of 58.583 and 20.452 mg g^–1^, respectively. The optimal extraction conditions involved acetonitrile for AV and ethanol for MB, with sample-to-adsorbent mass ratios of 1:5 and 1:1 (m/m), respectively, achieving recoveries higher than 80%. The adsorption kinetics followed a pseudo-second-order model, and the thermodynamic evaluation indicated a spontaneous process (Δ*G* ranging from −4.46 to −2.41 kJ mol^–1^ for MB and −4.84 to −4.46 kJ mol^–1^ for AV), consistent with physisorption mechanisms. Analytical validation demonstrated high sensitivity, with detection limits of 46.9 μg L^–1^ for AV and 4.6 μg L^–1^ for MB, and quantification limits of 156.3 and 15.2 μg L^–1^, respectively. Furthermore, the reusability evaluation showed excellent structural stability and multicycle performance, reinforcing the potential of the polymer as an environmentally friendly and low-cost solution for monitoring emerging contaminants in aquatic food products and contributing to food safety and environmental protection.

## Graphical abstract

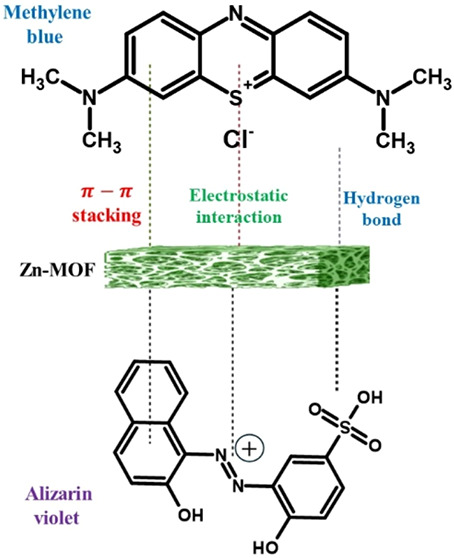

## Introduction

Dyes constitute one of the largest global industries, with significant economic and social ramifications in many countries.^1^ They are applied in various sectors, including textile processing, food production, and the pharmaceutical sector.^2^ Specifically, the textile industry produces an annual output of 700 000 tons of dyes and discharges approximately 2.15 billion liters of wastewater per year.^2^ Depending on factors such as fiber class, color, or industrial processes, there is a diverse range of dyes, which can pose significant pollution concerns if disposed of incorrectly.^1^ An estimated 80% of dye-laden wastewater is often discharged untreated into water bodies or used directly for irrigation, resulting in adverse effects on both human health and ecosystems.^2^

Food products derived from river and marine sources, including shrimp (*Macrobrachium acanthurus*), play a vital role in the diets of populations worldwide.^3^ However, the widespread application of synthetic dyes in aquaculture, fisheries, and agricultural activities near rivers has raised significant concerns about unintentional contamination of these food sources.^4^ Such contamination poses serious risks to human health, particularly through prolonged exposure or repeated ingestion, potentially leading to long-term adverse effects.^1^ Typically, synthetic dyes have carcinogenic, mutagenic, and teratogenic effects, posing toxic threats to plants, animals, and humans.

Among the dyes most used, alizarin violet (AV, sodium 4-hydroxy-3-[(2-hydroxynaphthalen-1-yl)­diazenyl]­benzenesulfonate) and methylene blue (MB, [7-(dimethylamino)­phenothiazin-3-ylidene]-dimethylazanium chloride) are highlighted.

AV (Figure 1A) is a synthetic dye that is characterized by a high chemical/biological oxygen demand, suspended solids in some cases, and an intense violet color.^5^ It finds wide applications in various sectors, including textiles, cosmetics, and food production. Similarly, MB (Figure 1B) is used as a fabric dye, imparting a rich blue color to clothing and textiles. Its versatility also extends to other industries, including photography, where it works as a colorant in certain types of films and photographic papers.^6^

**Figure 1:**
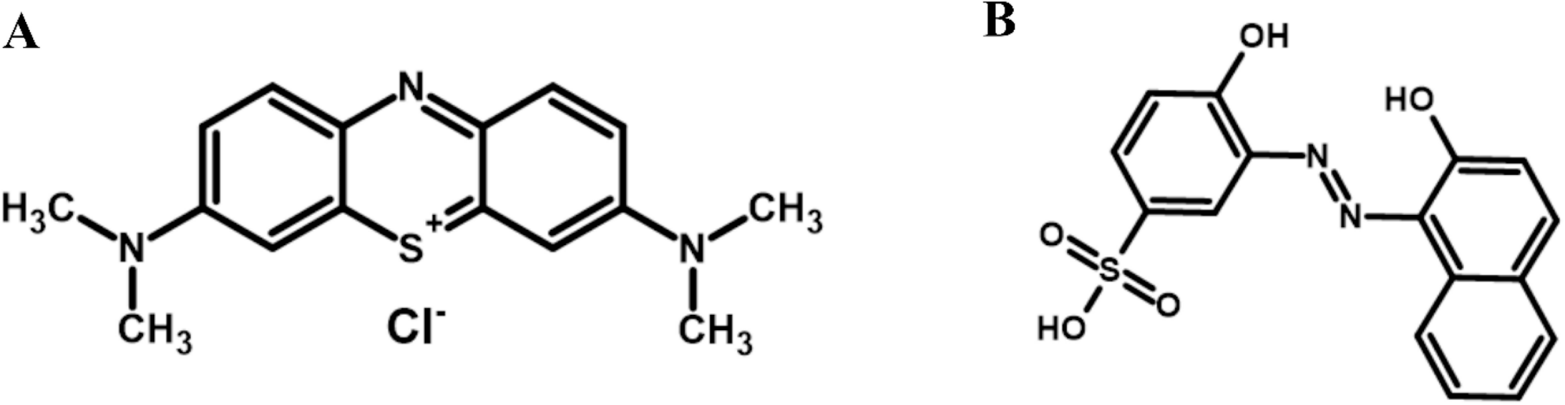
Molecular structures of the dyes investigated: (A) alizarin violet and (B) methylene blue.

Despite their widespread use and versatility, MB and AV pose environmental concerns.^1,2^ Like many synthetic dyes, their production and use can result in the contamination of waterways and ecosystems if not managed properly.^7^ For example, due to their recalcitrant nature characterized by a complex chemical structure containing five aromatic rings and two sulfonate groups, AV is notoriously difficult to degrade or remove from wastewater treatment processes, persisting in the environment.^5^ Their persistence not only poses challenges for environmental remediation but also raises concerns about their potential carcinogenic and toxic effects on living organisms.^8^

Based on this context, several methods for detecting these emerging contaminants, in diverse matrices, have been reported, including advanced oxidation processes (AOPs),^9^ biological processes,^10^ separation by membrane,^11^ and adsorption processes.^3^ Specifically, adsorption processes are widely recognized as effective strategies for extracting dyes from the environment, due to several advantages,^12^ including: (i) their remarkable effectiveness, (ii) cost-effectiveness, (iii) minimal generation of harmful byproducts, and (iv) low energy requirements.

In recent years, coordination compounds, or metal–organic frameworks (MOFs), have been used as high-performance adsorbents for dye removal due to their high surface area, porosity, chemical stability, and structural flexibility.^13,14^ For example, de Jesus et al.^15^ demonstrated the effectiveness of Ni-MOF in extracting various dye and pesticide residues from fish, achieving a recovery efficiency of more than 90%. Similarly, Daneshgar et al.^16^ synthesized UiO-66 and NH_2_–UiO-66 in different synthesis methods for removing dyes from wastewater, producing satisfactory results both in dye extraction and in the reuse of MOFs. In another study, Aljohani et al.^2^ reported the preparation of an environmentally acceptable Ag-MOF to remove dyes (malachite green) from industrial waters. In this study, the results demonstrated that the dye was mainly adsorbed on the Ag-MOF adsorbent by electrostatic attraction, hydrogen bonding, ion exchange, and pore-filling forces. Furthermore, it was found that the highest dye adsorption efficiency on the Ag-MOF adsorbent was 809.71 mg g^–1^.

Although existing literature recognizes the potential of MOFs in adsorption processes, there is a notable lack of studies focused specifically on their application in the analysis of food samples from water bodies, such as shrimp.^17^ Given this fact, this study presents a green approach based on multivariate analysis for extracting dyes (AV and MB) from shrimp samples.

The novelty of this work lies in the development of a sustainable strategy using [Zn­(Bz-COO)_2_]_∞_ for the removal of dyes from shrimp samples, offering a targeted approach to food safety and quality control. This study provides a comprehensive evaluation of the adsorption process, including thermodynamic parameters and kinetic factors such as the adsorption rate and mechanism. By focusing on food samples, it directly addresses a critical issue in food safety, paving the way for advanced analytical techniques that combine efficiency, sustainability, and practical relevance in real-world applications.

## Materials and Methods

### Reagents

Ethanol (EtOH), hexane (Hex), sodium hydroxide, and zinc nitrate hexahydrate (Zn­(NO_3_)_2_·6H_2_O) grade P.A were purchased from Synth (Brazil). HPLC grade acetonitrile (ACN) was purchased from Tedia (Fairfield, Ohio). Benzene-1,4-dicarboxylic acid (Bz-(COO)_2_) was obtained from Sigma-Aldrich (Germany). MB and AV standards were purchased from Vetec (Rio de Janeiro, Brazil). A Milli-Q system (Bedford) was used to obtain ultrapure water. All chemicals were used without additional purification steps. Cartridges (Luer Slip Descarpack, 5.0 mL) and glass wool (Perfyl Tech, Brazil) for sealing were used to perform the extractions.

### Solutions

Standard stock solutions with concentrations of 10.0 mg L^–1^ were prepared for each dye using ethanol. To prepare working standard solutions in the range of 40–1000 μg L^–1^, stock solutions were carefully diluted in ethanol. A calibration curve ranging from 40 to 800 μg L^–1^ was constructed for MB, while a broader range of 200–1000 μg L^–1^ was selected for AV. The concentration levels for each calibration curve were carefully chosen based on the lower detection limits specific to each analyte, ensuring accurate and reliable quantification across the respective ranges.

All solutions were stored at −18 °C. Physical-chemical properties of AV and MB are summarized in Table S1.

### Sample Collection

Shrimp samples (*M. acanthurus*, approximately 500.0 g) were obtained from a local market in Viçosa, Minas Gerais, Brazil. Samples were promptly refrigerated and transported to the laboratory under temperature-controlled conditions. Upon arrival, samples were processed in food processors, followed by immediate freezing at −20 °C. The frozen material was then dehydrated by freeze-drying and subsequently stored in hermetically sealed polyethylene bags.

### Preparation of [Zn­(Bz-COO)_2_]_∞_

The synthesis of [Zn­(Bz-COO)_2_]_∞_ was performed using a modified solvothermal method, as previously reported by our group.^15^ Initially, 0.4984 g of Bz-(COO)_2_ was dispersed in a beaker containing 10.0 mL of Type I water, followed by pH adjustment to pH 10 using NaOH solution (8.00 mol L^–1^) with stirring. Then, a total of 0.8925 g of Zn­(NO_3_)_2_·6H_2_O was slowly introduced into the solution while being mechanically stirred for 30 min. The prepared mixture was then placed in a 25.0 mL stainless steel autoclave, supplemented with 4.0 mL of ethylene glycol and subjected to heating at 200 °C for 24 h. Subsequently, the system was cooled naturally. The precipitate was separated by centrifugation (4000 rpm for 5 min). The resulting precipitate underwent two sequential washing steps using a 1:1 (v/v) mixture of deionized water and ethanol to remove impurities. The washed material was then dried in an oven at 60 °C for 4 h. The general synthesis procedure for [Zn­(Bz-COO)_2_]_∞_ is summarized in Figure S1.

### Characterization of [Zn­(Bz-COO)_2_]_∞_

A JEOL JSM-360-LV microscope operating at 20 kV with secondary electron imaging was used to obtain the scanning electron microscopy (SEM) images of the [Zn­(Bz-COO)_2_]_∞_. Sample holder coated with a 10 nm gold layer (Bal-tec MED020 sputter coating) was used to hold the samples. Furthermore, an FT-600 spectrometer (Agilent Technologies, United States) equipped with an ATR accessory was employed to perform group characterization functional within the [Zn­(Bz-COO)_2_]_∞_ structure. The mid-infrared range (400–4000 cm^–1^) was used to perform the measurements. For thermogravimetric analysis (TGA), a thermobalance (PerkinElmer, United States) was used. For this, 3.00 mg of [Zn­(Bz-COO)_2_]_∞_was positioned in sample holders and analyzed under nitrogen flow (50 mL min^–1^), with a heating program ranging from 25 to 900 °C at a rate of 10 °C min^–1^. Furthermore, the powder X-ray diffraction (PXRD) technique, using a Rigaku diffractometer, was used to evaluate the crystalline structures of the synthesized materials. The experiment was carried out at ambient temperature, scanning a 2θ range from 25 to 700 nm using Cu Kα radiation. Raman spectra were acquired using a Renishaw MicroRaman instrument (UK) equipped with a 785 nm laser source operating at 3 mW. Each measurement was performed with an integration time of 30 s, and the analysis was repeated five times to ensure accuracy and reproducibility.

### Brunauer–Emmett–Teller Analysis

To analyze the Brunauer–Emmett–Teller (BET) surface area and pore size distribution of [Zn­(Bz-COO)_2_]_∞_, a analyzer manufactured by Anton Paar (Austria) was applied. The analysis was conducted by nitrogen adsorption covering a relative pressure range of 0.0001 ≤ *P*/*P*_0_ ≤ 0.9918 at 77 K.^18,19^

### Point of Zero Charge (pH_PZC_) for [Zn­(Bz-COO)_2_]_∞_

To assess the point of zero charge (PZC) of [Zn­(Bz-COO)_2_]_∞_, a modified version of the procedure described by Vieira et al.^20^ was employed. Briefly, 10.0 mL solutions of 0.1 mol L^–1^ NaCl were prepared. The pH of each solution was adjusted using HCl (0.1 mol L^–1^) or NaOH (0.1 mol L^–1^) to achieve an initial pH range of 2 to 11. Subsequently, approximately 50.0 mg of [Zn­(Bz-COO)_2_]_∞_ was introduced into each solution. The mixtures were then gently agitated at 40 rpm for 24 h at room temperature. Then, the final pH of each solution was measured. ΔpH was determined using eq 1.

1 $$\Delta \mathrm{pH}={\mathrm{pH}}_{\mathrm{final}}-{\mathrm{pH}}_{\mathrm{initial}}$$

### Study of Adsorption Isotherm

Adsorption experiments were conducted following the method modified described by Carvalho et al.^3^ In this procedure, 100.0 mg of the adsorbent was added to 10.0 mL of diluted solutions with concentrations varying from 170 to 490 μg L^–1^ for both dyes (AV and MB). The solutions were stirred (40 rpm) for 24 h and kept at room temperature. Subsequently, the mixture was centrifuged at 4000 rpm and the supernatant concentration was determined using the maximum wavelengths of the dyes, λ_max_ = 501 nm and λ_max_ = 645 nm for AV and MB, respectively. For this assay, a calibration curve varying between 60 and 600 μg L^–1^ was prepared for both dyes. The amount of dye adsorbed at equilibrium (qe, mg g^–1^) was calculated using eq 2

2 $$qe=\frac{{(C}_{i}-C_{e})\times V}{m}$$

where, *C*_i_ and *C*_e_ are the initial concentration and equilibrium concentration, respectively, measured mg L^–1^. *V* represents the volume (L) of the standard solution (comprising dye), while “*m*” denotes the mass (mg) of the MOF used.

The experimental data were subjected to mathematical modeling. In this study, the Langmuir isotherm (eq 3) and the Freundlich isotherm (eq 4) were used.

3 $$\frac{C_{e}}{q_{e}}=\frac{1}{K_{L}\cdot q_{i}}+\frac{C_{e}}{q_{\max}}$$

4 $${\ln q}_{e}={\ln K}_{F}+\frac{1}{n}{\ln C}_{e}$$

In this context, *q*_max_ (mg g^–1^) denotes that the maximum adsorption capacity of *K*_L_ (L mg^–1^) is the Langmuir adsorption equilibrium constant, which refers to the adsorption energy. *K*_F_ (mg^1–1/*n*^L^1/*n*^g^–1^) is the Freundlich equilibrium constant, given insights into the adsorption capacity of the MOF. The parameter *n*, which is dimensionless, indicates the degree of heterogeneity. A value of *n* greater than 1 means strong interactions, whereas a value of *n* less than 1 indicates weaker interactions.

### Adsorption Kinetic Study

The adsorption kinetic was performed based on the methodology established by Vieira et al.^20^ In summary, about 100.0 mg of the MOF was added to a solution with 10.0 mL of dyes at a concentration of 350 μg L^–1^. The system was maintained under mechanical stirring at 250 rpm, and samples were taken at regular time intervals. After analysis, the samples were reintroduced into the system. The amount of adsorbed dye (*qt*, in mg g^–1^) at each point (*t*) was calculated using eq 5.

5 $$\mathrm{qt}=\frac{{(C}_{i}-C_{e})\times V}{m}$$

The adsorption kinetics of the dyes on [Zn­(Bz-COO)_2_]_∞_ were analyzed using both the pseudo-first order model (eq 6) and the pseudo-second order model (eq 7).

6 $${\mathrm{Ln}(q-q}_{t})={\ln q}_{e}-k_{1}t$$

7 $$\frac{t}{q_{t}}=\frac{1}{{{(K}_{2}\cdot t)}^{2}}+\frac{t}{q_{e}}$$

In the equations, *K*_1_ and *K*_2_ denote the parameters corresponding to the adsorption rate constant (g mg^–1^ min).

### Thermodynamic Study

To investigate the thermodynamic properties of the dyes-[Zn­(Bz-COO)_2_]_∞_ system, a concise study was conducted at temperatures of 281, 298, and 315 K as detailed by Silva et al.^21^ For this, 100.0 mg of the adsorbent was used in diluted 10.0 mL solutions with concentrations of 150 μg L^–1^ for both dyes (AV and MB). The mixture continuously agitated at 40 rpm for 24 h while maintained at ambient temperature. Then, the solutions were subjected to centrifugation at 4000*g*, and the concentration of the supernatant was analyzed by measuring the absorbance at the maximum wavelengths λ_max_ = 501 nm and λ_max_ = 645 nm for the dyes, AV and MB, respectively. The amount of dye adsorbed at equilibrium (*q*_e_, mg g^–1^) was calculated by using eq 2.

Thermodynamic parameters, such as changes in the free energy (Δ*G*°, kJ mol^–1^), enthalpy (Δ*H*°, kJ mol^–1^), and entropy (Δ*S*°, kJ mol^–1^K^–1^), were calculated.

Initially, the constant values (*K*_D_) were obtained by using eq 8. Δ*G*° (eq 9) was calculated at temperatures of 281, 298, and 315 K. Subsequently, a plot of the natural logarithm of the constant (ln *K*_D_) against the inverse of the temperature (1/*T*) was generated. According to the van’t Hoff eq (eq 10), the linear fit of this curve (the values of the angular and linear coefficients) provides the values of Δ*H*° and Δ*S*° involved in the adsorption processes studied.

8 $$K_{D}=\frac{q_{e}}{C_{e}}$$

9 $${\Delta G}^{{}^{\circ}}=\mathrm{RT}\;\ln\left(K_{D}\right)$$

10 $$\ln K=\frac{-\Delta H}{R}\left(\frac{1}{T}\right)+\frac{\Delta S}{R}$$

In this context, *R* represents the universal gas constant with a value of 8.314 J K^–1^ mol^–1^. T is the absolute temperature in Kelvin (*K*), and *K*_D_ is the equilibrium constant.

### Multivariate Analysis for Dye Extraction Using [Zn­(Bz-COO)_2_]_∞_

The extraction of the dyes (MB and AV) from shrimp samples was conducted using the matrix solid-phase dispersion (MSPD) technique, as previously described by our group.^22^ This method involves three steps, which include (i) homogenization of the solid sample with adsorbent, (ii) construction of the cartridge containing this homogenized mixture, and, subsequently, (iii) elution with a specific solvent. In this study, multifactorial analysis was carried out to determine the ideal conditions for extracting dyes from shrimp samples using [Zn­(Bz-COO)_2_]_∞_. Initially, a full factorial design (2^2^) was used. The factors evaluated were (i) the sample-to-adsorbent ratio (1:1; 1:5; 1:10 m/m) and (ii) the polarity of the eluent (EtOH, Hex, and ACN). The coded levels for the independent variables are detailed in Table 1. Model optimization and data fitting were performed by using central composite design (CCD) analysis with response surface methodology.

**Table 1:** Factors Assessed during the Multifactorial Analysis Using [Zn­(Bz-COO)_2_]_∞_ ^a^

		variable codes		
variables	symbols	–1	0	+1
adsorbent-to-sample ratio	*x*_1_	1:1	1:5	1:10
nature of the eluent	*x*_2_	Hex	ACN	EtOH

a ACN—acetonitrile; Hex—hexane; EtOH—ethanol.

The matrix (2^2^) is described in Table 2. To prevent bias, the analyses were conducted randomly.

**Table 2:** Coded Matrix for Multifactorial Analysis

run	eluent	ratio
1	–1	–1
2	–1	1
3	1	–1
4	1	1
5	–1	0
6	1	0
7	0	–1,414
8	0	1,4142
9 (C)	0	0
10 (C)	0	0

To evaluate the extraction efficiency, shrimp samples were enriched with standard solutions at a defined concentrations of 500 μg L^–1^ for dyes. Extraction efficiency was calculated using eq 11

11 $$\mathrm{extraction}\;\mathrm{efficiency}\left(\%\right)=\left(\frac{C_{i}-C_{f}}{C_{i}}\right)$$

Here, *C*_i_ is the initial concentration, and *C*_f_ is the final concentration.

### Sample Preparation Procedure

The extraction process followed the method described by de Carvalho et al.^15^ In summary, 0.10 g of shrimp was mixed with [Zn­(Bz-COO)_2_]_∞_ in masses ranging from 0.10 to 1.00 g for 3 min, corresponding to sample-to-adsorbent ratios of 1:1, 1:5, and 1:10 (m/m). The resulting mixture was then placed into a 5.00 mL polyethylene cartridge supported with glass wool. Elution was conducted under vacuum using 10.00 mL of an organic solvent (ACN, Hex, or EtOH). Then, the extracted solutions were filtered through a Nylon membrane filter (0.45 μm pore size, 4 mm diameter; Sartorius, Germany) before analysis. The dye extracts were then analyzed using a UV/vis spectroscopy at maximum wavelength of 501 nm for AV and 645 nm for MB. Efficiency of extraction of the dyes was calculated using eq 11.

### UV/vis Spectroscopy

To ensure accuracy in quantification, the extracted solutions were subjected to analysis using a Thermo Scientific Evolution Array model spectrophotometer (USA). The maximum absorbance wavelengths (λ_max_) were used to perform the quantification, being observed at λ_max_ = 501 and 645 nm for the AV and MB dyes, respectively. Linear regression analysis was used to generate calibration curves within the range of 40–1000 μg L^–1^, enabling subsequent validation and comparison.

### Method Validation

Several parameters were evaluated to assess the feasibility of the method, including accuracy, precision, selectivity, limit of detection (LOD), and limit of quantification (LOQ). Calibration curves were created using dilutions of standard solutions at a concentration of 10 mg L^–1^, covering a range of 40–800 μg L^–1^ for MB and 200–1000 μg L^–1^ for AV. Recovery percentages (%) were determined using eq 11. Enrichment factors were evaluated under ideal extraction parameters, with samples spiked at three concentration levels of the dyes: 300, 350, and 600 μg L^–1^. Furthermore, the method’s precision was assessed and reported as relative standard deviations (RPD).

### Statistical Treatment

Data processing for multivariate analysis and graphical representation was carried out using STATISTICA software (version 7). Analysis of Variance (ANOVA) and Tukey’s test were used to confirm significant differences between the assays, with a confidence level of 95%. Each experiment was conducted in triplicate to guarantee the robustness of the results obtained.

## Results and Discussion

### Characterization of [Zn­(Bz-COO)_2_]_∞_

After synthesis, the resulting product underwent comprehensive characterization using several techniques, including FT-IR, SEM, XRD, TGA, BET spectroscopy, and Raman spectroscopy.

The FT-IR spectrum of [Zn­(Bz-COO)_2_]_∞_ is illustrated in Figure 2A. Through a comparative analysis of the IR spectra of Bz-(COO)_2_ and [Zn­(Bz-COO)_2_]_∞_, it was possible to elucidate the coordination of the ligand to the Zn^2+^ ion. In the spectrum, notable changes were observed in the stretching vibrations for the ν­(C=O) and ν­(C–O) bonds, from 1648 to 1633 cm^–1^ and from 1204 to 1167 cm^–1^, respectively. Absorptions between 2964 and 2855 cm^–1^ corresponded to aromatic and aliphatic C–H stretching vibrations for both structures. Furthermore, absorptions between 729 and 696 cm^–1^ were indicative of the torsional vibrations of the aromatic ring for both structures. Within the range of 579–635 cm^–1^, we observed vibrations of the ν­(Zn–O) bonds. Notably, absorptions between 3500 and 3000 cm^–1^, characteristic of OH hydrogen bond stretching vibrations for BDC, were absent in the [Zn­(Bz-COO)_2_]_∞_. Possibly, these vibrations between 3500 and 3000 cm^–1^ are due to the presence of humidity.

**Figure 2:**
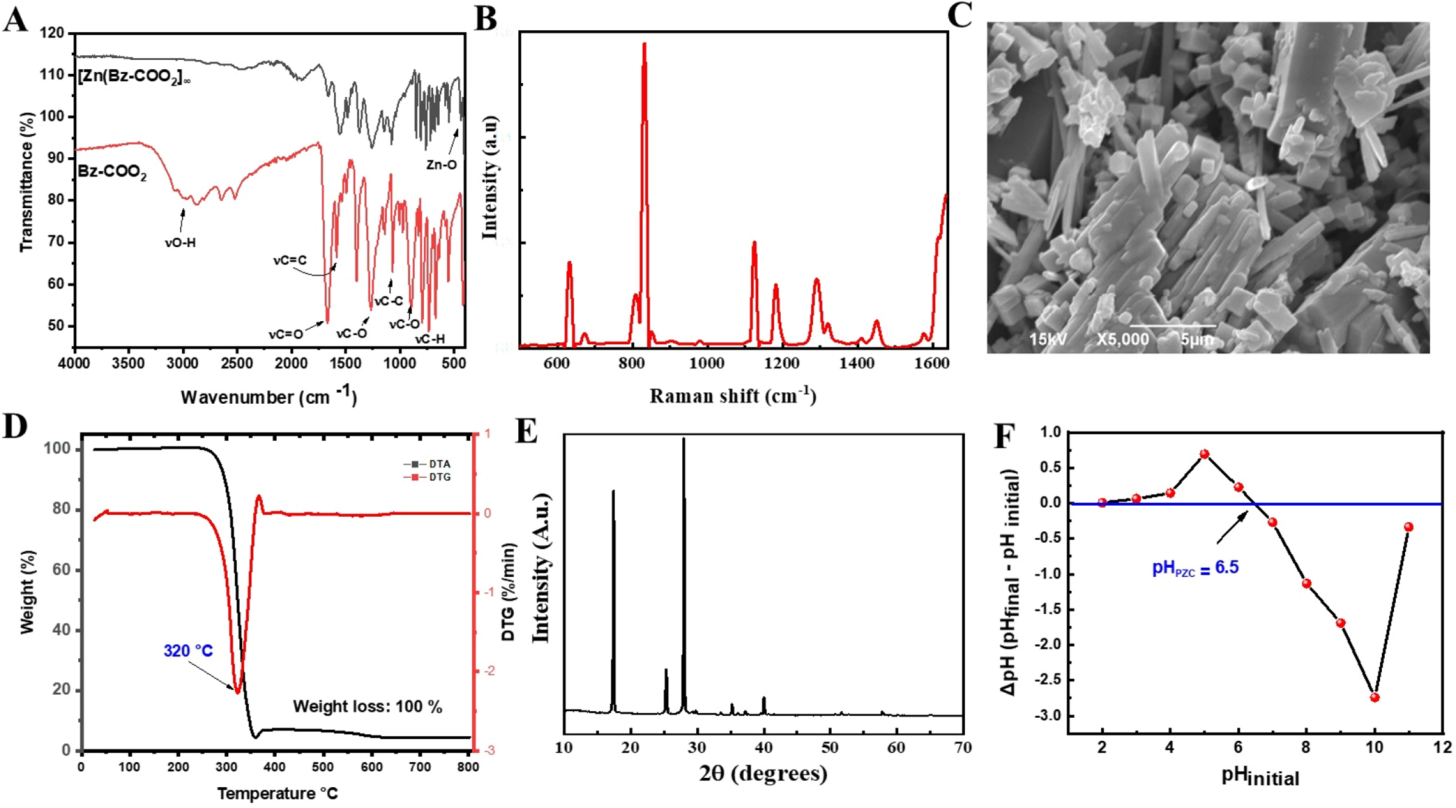
Comprehensive material characterization: (A) FT-IR analysis of the ligand (Bz-(COO)_2_) and the synthesized MOF, [Zn­(Bz-COO)_2_]_∞_; (B) Raman spectroscopy analysis; (C) scanning electron microscopy (SEM) images; (D) thermogravimetric analysis (TGA); (E) X-ray diffraction (XRD) patterns; and (F) determination of the point of zero charge (PZC).

Raman spectroscopy was employed to verify the successful synthesis of the [Zn­(Bz-COO)_2_]_∞_ sample. Figure 1B illustrates a representative room-temperature Raman spectrum, highlighting two distinct spectral regions at 500–1000 and 1400–1600 cm^–1^, each associated with specific molecular vibrations characteristic of the [Zn­(Bz-COO)_2_]_∞_ structure.^23^ In the higher frequency region, the main bands at 1542, 1466, and 1583 cm^–1^ correspond to well-defined molecular vibrations.^24^ The band at 1542 cm^–1^ is attributed to the symmetric stretching vibration of carboxylate group νCOO–, indicating successful coordination of the organic ligand with the Zn center. The band at 1466 cm^–1^ represents the νC–O stretching vibration of deprotonated hydroxyl groups, suggesting adequate deprotonation during the synthesis process.^23,24^ Finally, the 1583 cm^–1^ band is associated with in-plane deformation vibrations of the benzene rings in the organic linker, which is a critical structural component of the [Zn­(Bz-COO)_2_]_∞_ structure. In the lower frequency range, the bands at approximately 614 and 816 cm^–1^ likely arise from bending and deformation vibrations of the benzene ring, indicative of interactions between the benzene rings and the metal center. These vibrational modes confirm the stability and integrity of the benzene ring structure within the [Zn­(Bz-COO)_2_]_∞_. Together, these spectral features serve as a molecular fingerprint, validating the successful synthesis and structural integrity of the [Zn­(Bz-COO)_2_]_∞_ material.^23,24^

SEM was employed to investigate the morphology of [Zn­(Bz-COO)_2_]_∞_, providing detailed information about its structural features. As illustrated in Figure 2C, the prepared MOF exhibits a well-defined rod-shaped morphology indicative of its highly organized crystalline nature. The rods are uniform in size and shape, suggesting a controlled synthesis process. Furthermore, SEM images reveal a high degree of porosity, with visible surface cavities and interstitial spaces that are characteristic of MOF structures. This porosity is a key feature, as it directly influences the adsorption properties of the material and its potential applications in processes such as dye removal.^25^ The observed morphology highlights the effectiveness of the synthesis method used, ensuring the formation of a material with optimized structural and functional properties.

The thermal stability of [Zn­(Bz-COO)_2_]_∞_ was evaluated at temperatures of up to 900 °C under a nitrogen atmosphere.^26,27^ The results revealed that synthesized [Zn­(Bz-COO)_2_]_∞_ exhibited hydrophobic characteristics, with notable mass loss observed at 320 °C, as depicted in Figure 2D. This thermal decomposition event corresponded to complete degradation of the ligand (BDC), leading to the formation of the ZnO residue. The observed high thermal stability of the synthesized material suggests its potential suitability for a wide range of applications, including adsorbent for contaminant extraction. This characteristic is particularly valuable in environments with high temperatures or when subjected to thermal processing steps.

The material [Zn­(Bz-COO)_2_]_∞_ was also subjected to XRD analysis to determine its structure and crystallinity. The XRD pattern of synthesized [Zn­(Bz-COO)_2_]_∞_ is shown in Figure 2E. Intense peaks observed at small angles (2θ) suggest the presence of abundant pores in the structure of the material.^28,29^ Furthermore, sharp peaks detected at 2θ = 18, 25, and 28° indicate that the crystallinity of [Zn­(Bz-COO)_2_]_∞_ is substantial.

Furthermore, by examination of the pH_PZC_, as illustrated in Figure 2F, it was noted that the material exhibits a PZC at pH 6.5. At this pH, there is an equilibrium between the positive and negative charges on the nanomaterial surface. Consequently, above pH 6.5, the surface of the material becomes negatively charged, while below pH 6.5, it becomes positively charged. This characteristic facilitates the extraction of analytes, highlighting the importance of pH adjustment in optimizing adsorption processes.^30^

### BET Analysis

BET measurement was conducted to explore the surface area and porosity characteristics of [Zn­(Bz-COO)_2_]_∞_. Nitrogen adsorption–desorption isotherms were recorded at 77 K, and the resulting curves, depicted in Figure 3A, indicated that [Zn­(Bz-COO)_2_]_∞_ presents a type III isotherm.^19^ Based on the BET model, the material exhibited a surface area of 5216 m^2^/g and a total pore volume of 0.0136 cm^3^ g^–1^ Furthermore, the average pore diameter was determined to be 17.34 nm, confirming the microporous nature of [Zn­(Bz-COO)_2_]_∞_. Figure 3B illustrates the pore size curves of [Zn­(Bz-COO)_2_]_∞_.

**Figure 3:**
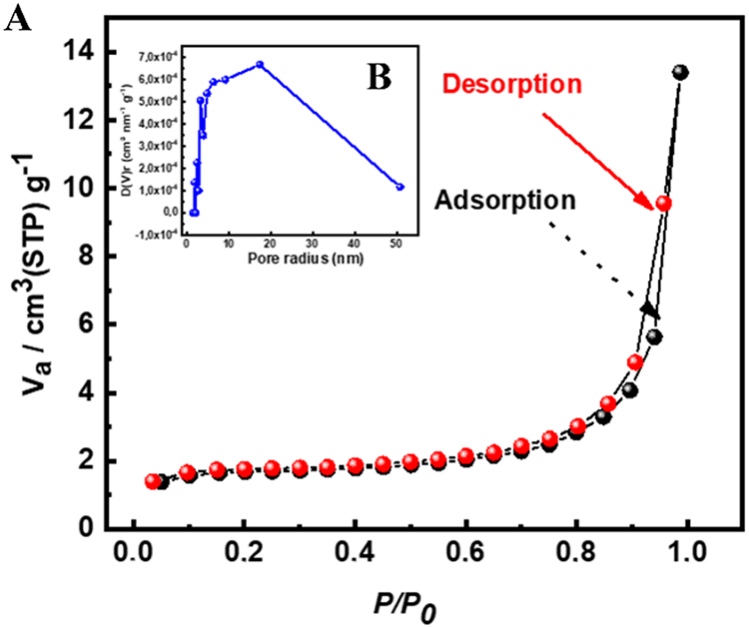
BET analysis results: (A) adsorption–desorption isotherm and (B) pore size distribution, illustrating material surface area and porosity characteristics.

### Multivariate Analysis Using [Zn­(Bz-COO)_2_]_∞_

To optimize dyes extraction using [Zn­(Bz-COO)_2_]_∞_, an multifactorial analysis was used, employing a full factorial design (2^2^) followed by CCD. The response variable used for optimization was recovery efficiency (%). The MSPD method was used to extract the dyes from the shrimp sample. Two main factors were examined: (i) the polarity of the extraction solvent, categorized as low, medium, and high, employing Hex, EtOH, ACN; and (ii) the ratio of sample-to-adsorbent, tested at 1:1, 1:5, and 1:10 (w/w). The findings of the optimization are presented in Table S2 for AV and Table S3 for MB. Figure 4A,B illustrates the response surface and levels curve, respectively, for AV extraction, while Figure 4C,D demonstrates the same for MB. These figures show the ideal conditions required to attain the highest recovery rates. As showed in Figure 4, the most favorable conditions for achieving satisfactory recovery (>80%) for AV and MB involved using ACN and EtOH, respectively, as the elution solvents, along with a sample-to-adsorbent mass ratio of 1:5 for AV and 1:1 for MB.

**Figure 4:**
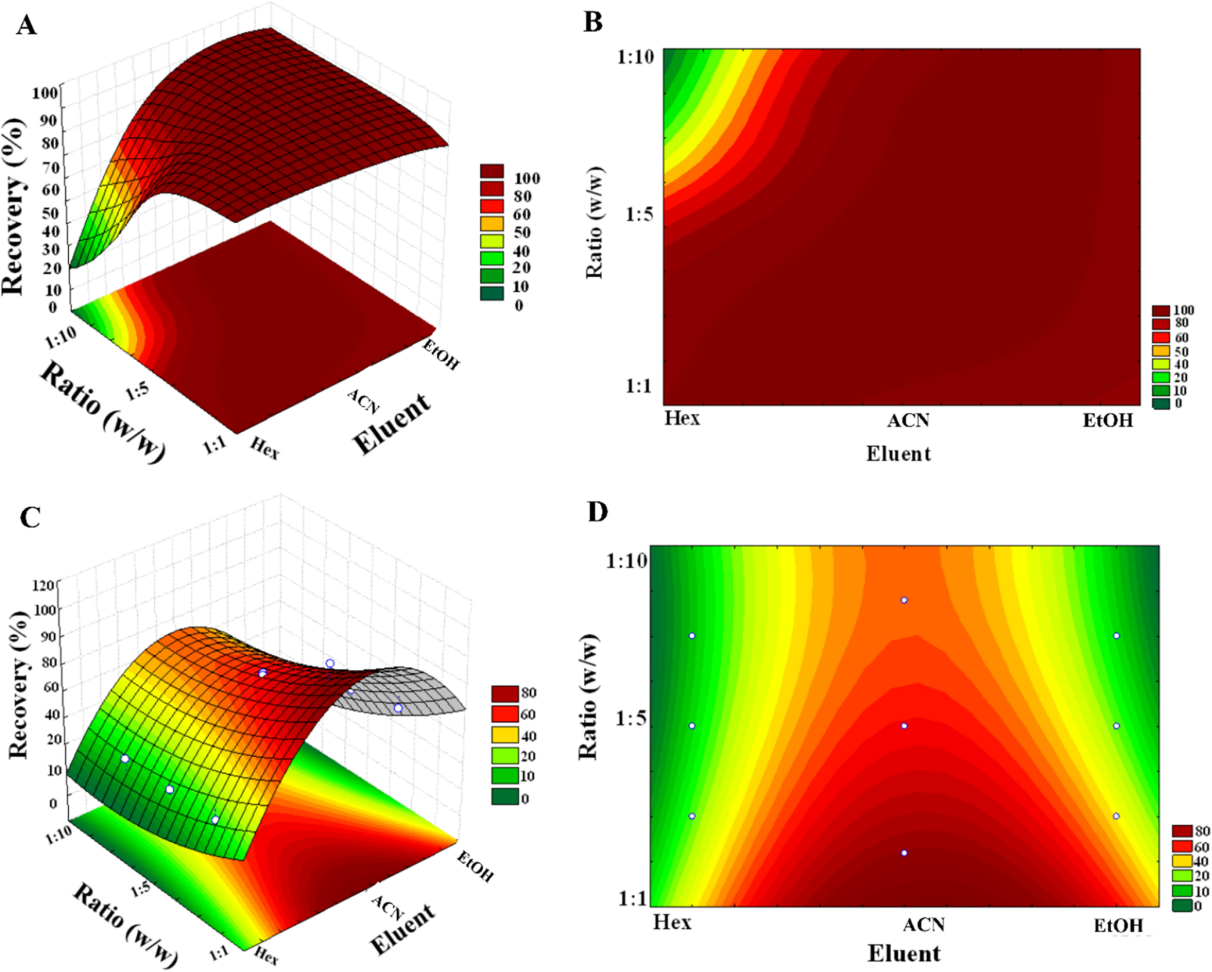
Response surface and contour analysis: (A) Response surface and (B) contour plot for AV; (C) response surface and (D) contour plot for MB, demonstrating the interaction effects of the studied variables on dye adsorption.

The quadratic polynomial model represents the relationship between dye extraction efficiency and the factors within the tested ranges and can be represented by eqs 12 and 13, as follows

12 $$Y_{\mathrm{VA}}=15.83+28.16x_{1}+16.88{x_{1}}^{2}+4.29y+0.68{x_{2}}^{2}+12x_{1}x_{2}+0.014$$

13 $$Y_{\mathrm{MB}}=61.03+6.33x_{1}-51.551{x_{1}}^{2}-12.22x_{2}+3.28{x_{2}}^{2}-3.75x_{1}x_{2}+0.021$$

Equations formulated in terms of coded factors (*x*_1_ e *x*_2_) allow the prediction of reactions at specific levels of each factor. For VA, it was noted that the nature of the eluent significantly impacted (*p* < 0.05) its extraction using [Zn­(Bz-COO)_2_]_∞_. In this case, when the polarity is increased, a higher VA recovery rate is consistently achieved at any adsorbent-to-sample ratio (Figure 4A,B). On the other hand, when the polarity is decreased, a lower dye recovery rate is observed. In relation to AM, both factors (eluent and adsorbent/sample ratio) were significant (*p* < 0.05). In this proposed model, the adsorbent-to-sample ratio was found to notably influence dye extraction; however, its effect decreases when low and high polarity eluents are used (Figure 4C,D). Notably, in medium polarity eluents, recovery was greater than 70%.

The quality of the data fit was evaluated through ANOVA. Statistical parameters obtained from ANOVA for the extraction of AV and MB dyes via multivariate optimization are summarized in Table 3.

**Table 3:** ANOVA Data for Multifactorial Analysis of Dye Extraction Utilizing [Zn­(Bz-COO)_2_]_∞_ ^a^

central composite design parameters							
	sum quadratic (*S*)			*F*_calc_		*p*	
factor	AV^b^	MB^c^	degrees of freedom	AV^b^	MB^c^	AV^b^	MB^c^
*x*_1_	4760.16	248.89	1	24.68	185.87	<0.05	<0.05
*x*_2_	145.96	1177.55	1	0.012	879.41	>0.05	<0.05
interaction *x*_1_ vs *x*_2_	576.00	52.35	1	2.99	39.098	>0.05	>0.05
pure error	771.29	1.339	6				
residue	6922.50	8791.03	15				
*R* ^2^	0.888	0.939					
*R* adjusted	0.749	0.8634					

a *x*_1_—eluent; *x*_2_—adsorbent:sample ratio (w/w).

b Alizarin violet.

c Methylene blue.

Observing the coefficient of determination (*R* ^2^) for both dyes in the Table 3, it is evident that the model presents a strong fit to the data, with *R* ^2^ values of 0.888 for AV and 0.939 for MB. The Pareto chart (Figure S2) shows the importance of factors and their impact on the extract process of dyes. In Figure 4A, it is evident that both the nature of the eluent and the adsorbent:sample ratio demonstrate a positive linear influence on the extraction efficiency of dyes in the shrimp sample. Furthermore, Figure S3 effectively illustrates the data fit by comparing experimental data to predicted values. The proximity of the points to the red line indicates a superior fit of the data, suggesting the fit of the data to the model and confirming the optimal extraction conditions for both dyes.

### Adsorption Isotherms

Data obtained from adsorption experiments were used to fit the mathematical models, specifically, Langmuir and Freundlich. This approach aimed at exploring the adsorption behavior of the dyes on the [Zn­(Bz-COO)_2_]_∞_ surface. The adsorption capacity exhibited an increasing trend as the dye concentration increased. Figure 5A,B, together with Table 4, show the results of fitting the experimental data to the Langmuir and Freundlich adsorption models.

**Table 4:** Parameters Derived from Adsorption Experiments

		analytes	
	parameters	AV^a^	MB^b^
Langmuir	*R* ^2^	0.932	0.933
	*q*_max_ mg g^–1^	2.045	5.858
	*K*_l_ L mg^–1^	0.990	0.266
Freundlich	*R* ^2^	0.995	0.934
	*K*_f_ mg^1–1/*n*^L^1/*n*^g^–1^	1.491	0.227
	*n*	7.96	1.251
pseudo-first-order	*q*_e_ (mg g^–1^)	0.255	0.275
	*K* (1/min)	0.015	0.291
	*R* ^2^	0.908	0.825
pseudo-second-order	*q*_e_ (mg g^–1^)	0.307	0.287
	*K* (1/min)	0.058	1.242
	*R* ^2^	0.932	0.848

a Alizarin violet.

b Methylene blue.

**Figure 5:**
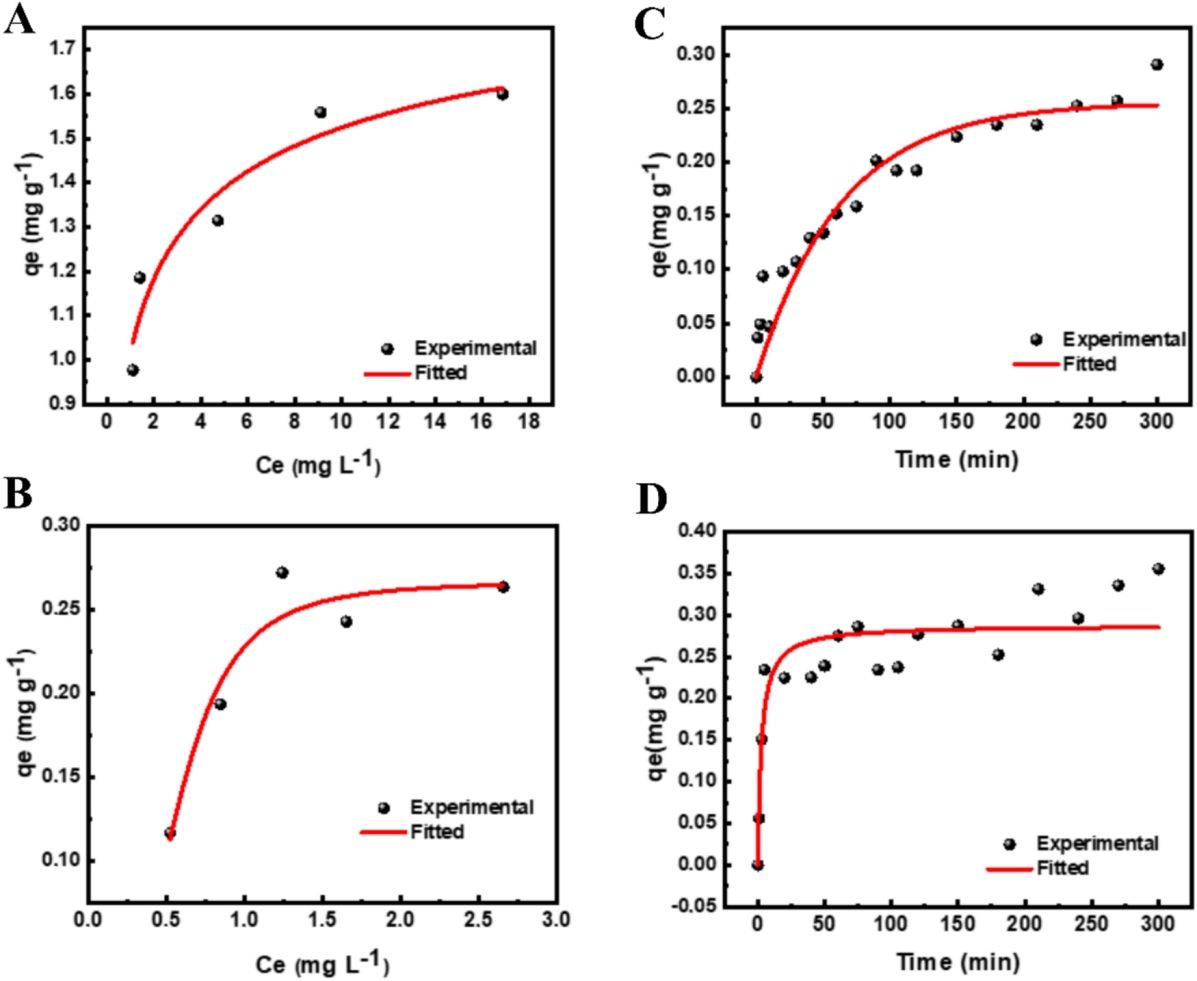
Adsorption isotherm and kinetics studies: (A) Adsorption isotherm for AV; (B) adsorption isotherm for MB; (C) adsorption kinetics for AV; (D) adsorption kinetics for MB.

Notably, the Freundlich model, with *R* ^2^ values ranging from 0.995 to 0.934 for AV and MB, respectively, showed superior linear fitting results compared to the Langmuir model, with *R* ^2^ values ranging from 0.932 to 0.933 for AV and MB, respectively. The static adsorption process of [Zn­(Bz-COO)_2_]_∞_ on dyes is characterized by a single-molecule-layer adsorption mechanism. Calculations based on the Langmuir model revealed maximum adsorption capacities of [Zn­(Bz-COO)_2_]_∞_ for AV and MB of 5.8583 and 2.0452 mg g^–1^, respectively.

### Adsorption Kinetic

The investigation of the adsorption mechanism between [Zn­(Bz-COO)_2_]_∞_ and dyes (AV and MB), along with the determination of the rate-determining steps in the adsorption process, involved the use of pseudo-first-order and pseudo-second-order kinetic models. The resulting adsorption kinetic data were then subjected to fit analysis. Figure 5C,D illustrates that the adsorption capacity of [Zn­(Bz-COO)_2_]_∞_ for dyes improves with increasing adsorption time. When examining the data presented in Table 4 and the fitted results represented in Figure 5C for AV and Figure 5D for MB, it becomes evident that the pseudo-second-order kinetic model with *R* ^2^ values of 0.932 for AV and 0.848 for MB exhibited better linearity compared to the pseudo-first-order kinetic model, with *R* ^2^ values of 0.908 for AV and 0.825 for MB. This suggests that the adsorption of dyes can occur in at least two steps, one slow and the other faster and that it depends both on the amount of [Zn­(Bz-COO)_2_]_∞_ and on the amount of dye present in solution.

### Thermodynamic Study

Thermodynamic data were obtained from temperature gradient experiments (Figure 6). These experiments allowed us to plot the slope and intercept of the ln *K*_d_ versus 1/*T* curve (Figure S4) which were useful for calculating the values of Δ*H* and Δ*S*. Table 5 presents the thermodynamic parameters obtained.

**Table 5:** Thermodynamic Parameters for the Interaction between [Zn­(Bz-COO)_2_]_∞_ and the Dyes

			Δ*G°* (kJ mol^–1^)			
dye	Δ*H°* (kJ mol^‑1^)	Δ*S°* (kJ mol^‑1^ K^‑1^)	281 K	298 K	315 K	*R* ^2^
MB^a^	–13444.1	–0.1358	–7.01	–4.46	–2.41	0.99
AV^b^	–1921.3	–0.0058	–4.46	–4.84	–4.53	0.92

a Alizarin violet.

b Methylene blue.

**Figure 6:**
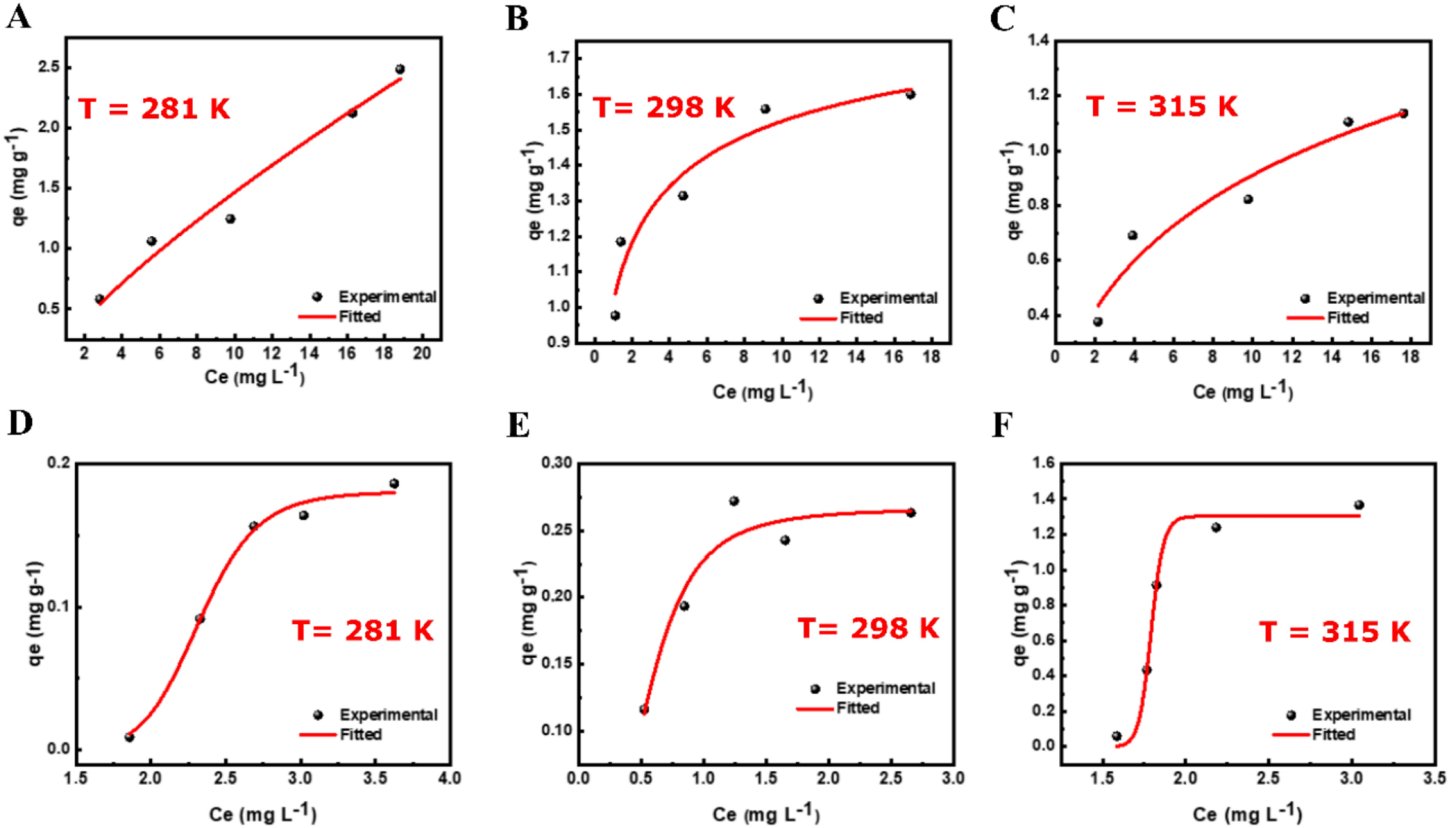
Thermodynamic study of the interaction between [Zn­(Bz-COO)_2_]_∞_ and dyes at different temperatures: (A–C) AB; (D–F) MB.

From Table 5, it is observed that the Δ*H* enthalpy for the adsorption of both dyes by [Zn­(Bz-COO)_2_]_∞_ was negative, with values of −13444.1 kJ mol^–1^ for MB and −1912.3 kJ mol^–1^ for AV. This result suggests that the adsorption of dyes to the MOF is an exothermic process with energy release. Furthermore, the negative value of the entropy change (Δ*S*) reveals the increasing randomness at the solid/solution interface. For Δ*G*, negative values were obtained at all temperatures for both dyes, ranging from −4.46 to −2.41 kJ mol^–1^ for MB and from −4.84 to −4.46 kJ mol^–1^. This fact suggests that the adsorption process of both dyes (MB and AV) on the [Zn­(Bz-COO)_2_]_∞_ surface is viable and spontaneous.

### Adsorption Mechanism Involving [Zn­(Bz-COO)_2_]_∞_

Based on the findings described in the Thermodynamic Study section, it was observed that the adsorption process of AV and MB on the MOF surface is a spontaneous exothermic physicochemical phenomenon. One of the driving forces underlying the interaction between the dyes and the MOF appears to be electrostatic attraction. This inference arises from the conditions of the medium (pH 7.4) during the adsorption process, where the dyes were fully deprotonated (see p*K*_a_ of dyes in Table S1), resulting in an overall negative charge, while the material exhibited an overall positive charge, thus facilitating the interaction between the adsorbate and the adsorbent. However, it is hypothesized that other forms of interactions also contribute to the effectiveness of the adsorption process. These may include hydrogen bonds and van der Waals forces.^7^ Figure S5 shows the FTIR spectra corresponding to the pure [Zn­(Bz-COO)_2_]_∞_ material (Figure S5A), as well as after the adsorption process of the MB (Figure S5B) and AV (Figure S5C) dyes. Comparative analysis of the spectra allows us to identify interactions between the functional groups present in the dyes and the active sites of [Zn­(Bz-COO)_2_]_∞_, indicating structural changes that suggest successful adsorption.

After MB adsorption, the spectrum (Figure S5B) reveals significant changes in the 1400–1500 cm^–1^ regions related to the vibrations of N–C of the dye. Furthermore, a shift or reduction in the intensity of the MOF carboxylate bands (1650–1500 cm^–1^) is observed, indicating possible electrostatic interactions or hydrogen bonding between the dye and the active sites of [Zn­(Bz-COO)_2_]_∞_. In the AV spectrum (Figure S5C), the main changes occur in the regions of 3300 cm^–1^ and 1650 cm^–1^, attributed to O–H and C=O vibrations, respectively, suggesting the formation of bonds between the phenolic and/or quinonoid groups of the AV molecule and the MOF. Furthermore, changes in the range of 1300–1100 cm^–1^, associated with C–O vibrations, are observed. These band changes evidence that the material [Zn­(Bz-COO)_2_]_∞_ interacts with the dyes, promoting adsorption through electrostatic interactions, π–π stacking, and hydrogen bond formation.

In a similar study, Adesina Adegoke et al.^31^ demonstrated that a zinc (Zn­(II))-based porous MOF decorated with carboxylate groups exhibited extremely high and rapid dye adsorption. This behavior can be attributed to specific interactions between the dye molecules and the MOF surface. The carboxylate groups play an essential role by acting as active sites for intermolecular interactions such as hydrogen bonding and electrostatic interactions. In addition, the high porosity of the material contributes to a significant increase in the available surface area, allowing for greater accessibility to the active sites and promoting rapid capture of the dye molecules. The presence of the Zn­(II) metal ion can also facilitate coordination interactions with certain functional groups of the dyes, further enhancing the adsorption efficiency.^31^

### Figure of Merit of the Method, Employing [Zn­(Bz-COO)_2_]_∞_ in the Extraction of Dyes

In this study, the effectiveness of the developed method was evaluated although several performance parameters, including working range, linearity, LODs, LOQs, accuracy, and precision. The values obtained are summarized in Table 6.

**Table 6:** Performance Parameters for the Proposed Dye Extraction Method Employing [Zn­(Bz-COO)_2_]_∞_

		calibration data			
analyte	linear range (μg L^–1^)	calibration equation	*R* ^2^	LOD (μg L^–1^)	LOQ (μg L^–1^)
AV^a^	200–1000	*y* = 0.0321 + 0.0032	0.998	46.9	156.3
MB^b^	40–800	*y* = 0.2084*x* – 0.0029	0.997	4.6	15.2

a Aizarin violet.

b Mthylene blue.

Analysis of Table 6 reveals satisfactory linearity for both dyes, covering a working range from 200 to 1000 μg L^–1^ with *R* ^2^ = 0.998 for AV, and a working range from 40 to 800 μg L^–1^ with *R* ^2^ = 0.997 for MB. The LOD and LOQ for AV were 46.9 and 156.3 μg L^–1^, respectively, while for MB, LOD, and LOQ were 4.6 and 15.2 μg L^–1^, respectively. To evaluate the accuracy of the method, spiked samples were analyzed in triplicate (Table 7). RSDs varied from 1 to 6% (*n* = 3). The findings demonstrate that the proposed method exhibits strong linearity, high sensitivity, and excellent precision, establishing it as an alternative approach to extract and quantify AV and MB in shrimp samples.

**Table 7:** Accuracy of Dye Recovery from Fortified Shrimp Samples

	concentration (μg L^–1^)		
analyte	added	found (mean ± SD)	recovery (%, ±RSD^a^)
AV^a^	300	243 ± 6	81 ± 2
	450	387 ± 5	86 ± 1
	600	533 ± 4	89 ± 1
MB^b^	350	315 ± 18	90 ± 6
	450	429 ± 19	95 ± 4
	600	564 ± 18	94 ± 3

a Aizarin violet.

b Mthylene blue.

### Determination of AV and MB Dyes in Real Samples

Shrimp samples from markets in Viçosa, Minas Gerais, Brazil were used to analyze dye residues using the proposed method. From analytical measurements, it was observed that the concentration obtained from the commercial sample was below the LOD of the proposed method.

### Reusability Studies

The synthesized material, [Zn­(Bz-COO)_2_]_∞_, was evaluated for its stability and extraction efficiency over multiple reuse cycles. These factors were evaluated by monitoring the dye recovery over three consecutive cycles of adsorption and desorption, using the same material. Considering the chemical stability of the MOF, desorption was performed under ultrasonic agitation for 15 min using a solution containing nitric acid (1:1 v/v) and hydrogen peroxide (30%, v/v) to facilitate analyte removal. After this process, the material underwent thorough washing with a water/ethanol solution (1:1, v/v) followed by drying at 40 °C for 3 h to prepare it for reuse. The results demonstrated satisfactory recovery rates exceeding 70%, for up to five recycling cycles, as shown in Figure S6. This performance highlights the robustness of the material and confirms its effective reusability in multiple cycles, supporting the recyclability and sustainability of [Zn­(Bz-COO)_2_]_∞_.

### Comparison with Other Methods

The performance of the synthesized [Zn­(Bz-COO)_2_]_∞_ adsorbent was compared with other adsorbent materials, as summarized in Table 8. Key parameters, including adsorption capacity, stability, and reusability, demonstrate that [Zn­(Bz-COO)_2_]_∞_ offers a competitive advantage in terms of dye removal efficiency and sustainable reusability over multiple cycles. Notably, the adsorbent achieved satisfactory recovery rates (>70%) up to five cycles, a performance that aligns well with green chemistry. This comparison highlights the [Zn­(Bz-COO)_2_]_∞_ material as an efficient and robust alternative for dye adsorption, combining high efficiency and practical reusability.

**Table 8:** Comparison of the Efficiencies of MOFs for Dye Extraction from Complex Samples^a^

MOF	cycle	recovery (%)	*q*_max_ (mg g^–1^)	reference
Fe_3_O@Ag-MOF	6	>92	1.68	^32^
MOF-808	4	>97	>633	^33^
MOF-5/BiCoO_3_	5	>90		^34^
MOF-Fe	4	>35	>21	^35^
Fe_3_-NH-MIL-88B		>77	280	^36^
[Ni(BDC)]_*n*_	3	>98	4.88	^15^
[Zn(Bz-COO)_2_]_∞_	5	>80	58	this work

a *q*_max_—maximum adsorption capacity.

## Conclusions

[Zn­(Bz-COO)_2_]_∞_ was successfully synthesized and characterized, confirming its stability and eco-friendly properties. The adsorption studies highlighted its strong interaction with AV and MB dyes, with maximum adsorption capacities of 5.8583 and 2.0452 mg g^–1^, respectively, as determined by the Langmuir model. Optimal recovery conditions (>80%) were achieved using ACN and EtOH as elution solvents for AV and MB recovery, respectively. Kinetic evaluations revealed that the pseudo-second-order model effectively described the adsorption process, while thermodynamic analysis demonstrated that the adsorption was spontaneous, with negative Δ*G* values across all temperatures for both dyes. The developed method showed excellent sensitivity, with LOD and LOQ values as low as 4.6 and 15.2 μg L^–1^ for MB. These findings confirm the potential of [Zn­(Bz-COO)_2_]_∞_ as a sustainable and recyclable adsorbent for the efficient extraction and determination of contaminants in complex samples, promoting eco-friendly analytical applications.

## Supplementary Material



## Notes

(i) Chemical reaction scheme for obtaining [Zn­(Bz-COO)_2_]_∞_; (ii) tables and figures related to the optimization of the extraction process; (iii) thermodynamic study graphs; (iv) FT-IR spectra of the material [Zn­(Bz-COO)_2_]_∞_ after dye extraction; and (v) reuse study graph of [Zn­(Bz-COO)_2_]_∞_ (PDF)
