# Supplementary material for: Multivariate Optimization of an Eco-Friendly Zinc-Based MOF for Adsorptive Removal of Emerging Contaminants in Food Samples
Source: ACS Omega. 2025 Sep 11;10(37):42901–12. doi: 10.1021/acsomega.5c05658 (PMC12461301; doi:10.1021/acsomega.5c05658)
Supplement: Supplementary file 1 [file ao5c05658_si_001.pdf]

Supplementary Material

Multivariate optimization of an ecofriendly zinc-based MOF for adsorptive removal of emerging contaminants in food samples

Wesley C. P. Aquino[a], Iare S. Ribeiro[a], Marcos V. S. Pereira[a], Luciano M. Guimarães[b], Gilberto R. da Silva-Junior[b], Renê C. da Silva[b], Jemmyson R. de Jesus[a*]

[a] Research Laboratory in bionanomaterials, LPbio, Department of Chemistry, Federal University of Viçosa, 36570-900, Viçosa, Minas Gerais, Brazil.

[b] Department of Physics, Federal University of Viçosa, 36570-900, Viçosa, Minas Gerais, Brazil

*Corresponding author:

Prof. Jemmyson R. de Jesus

e-mail address: jemmyson.jesus@ufv.br

Table S1. Physical-chemical properties of the dyes

Dyes	Molecular Weight (g mol^{-1})	Molecular Formula	Chemical class	Solubility	pKa
**Alizarin Violet**	366.3	$C_{16}H_{11}N_2NaO_5S$	Azo dyes	Water and alcohol	4-5
**Methylene Blue**	319.8	$C_{16}H_{18}N_3S.Cl$	thiazine group	Water and alcohol	5-6

Table S2. Recovery efficiency (%) of alizarin violet after multivariate optimization

Run	Eluent	Adsorbent/sample ratio	Recovery (%)
1	-1	-1	5.0
2	-1	1	5.0
3	1	-1	44.0
4	1	1	92.0
5	1	1	5.0
6	1	0	48.0
7	0	-1.414	19.0
8	0	1.414	9.0
9 (C)	0	0	20.0
10 (C)	0	0	10.0

Table S3. Recovery efficiency (%) of methylene blue after multivariate optimization

Run	Eluent	Adsorbent/sample ratio	Recovery (%)
1	-1	-1	6.0
2	-1	1	5.0
3	1	-1	26.0
4	1	1	10.0
5	1	1	5.0
6	1	0	18.0
7	0	-1.414	97.0
8	0	1.414	40.0
9 (C)	0	0	61.0
10 (C)	0	0	59.0

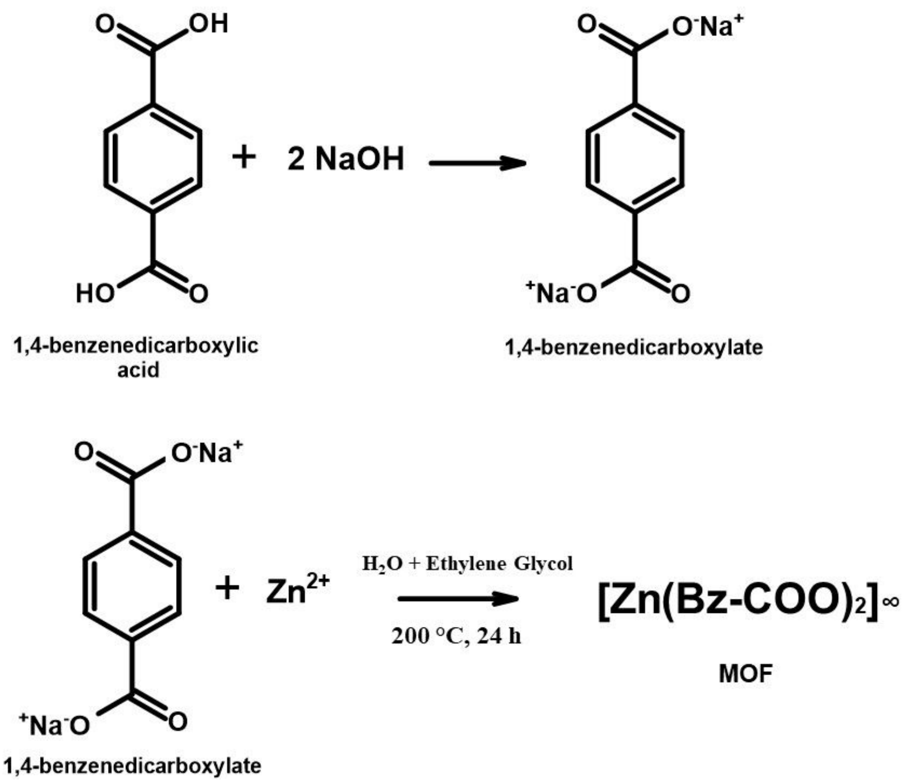

Figure S1. Scheme of chemical reaction that occurred in two steps to obtain $[Zn(Bz\text{-}COO)_2]_\infty$

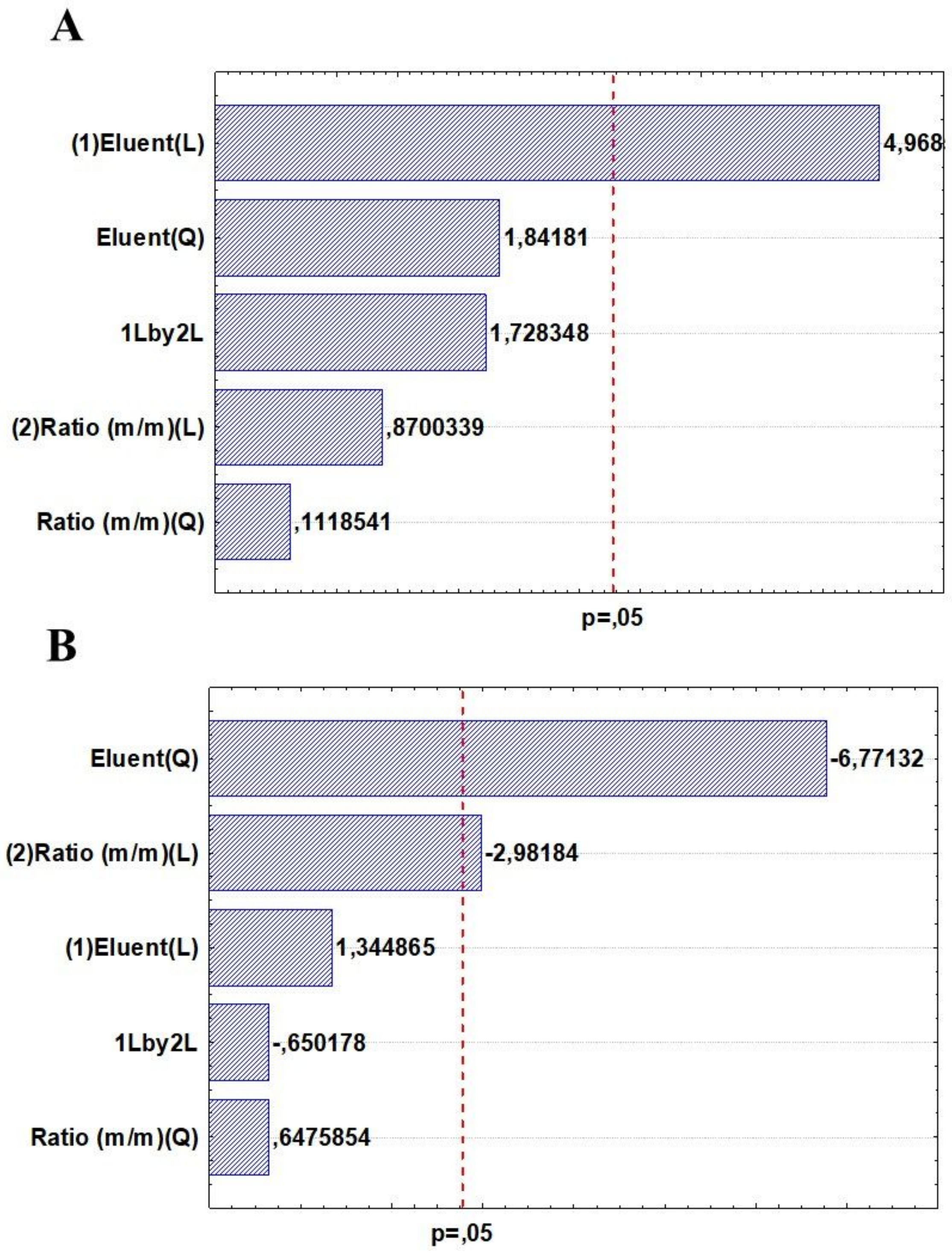

Figure S2. Pareto Chart for **(A)** Alizarin Violet and **(B)** Methylene Blue illustrating the factors that significantly affect (<0.05) the extraction process.

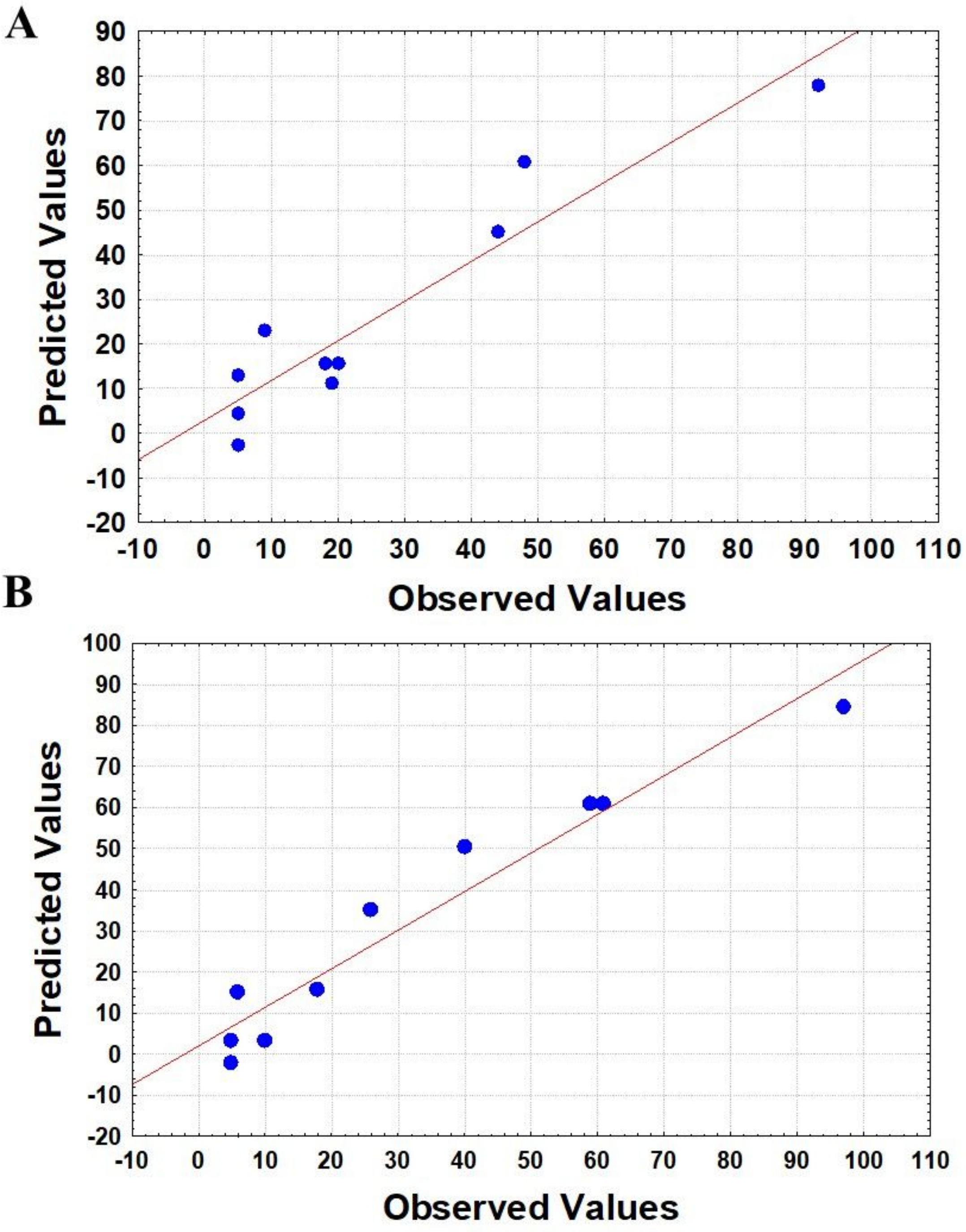

Figure S3. Experimental data versus predicted values for Alizarin Violet **(A)** and Methylene Blue **(B)**, demonstrating the fit of the data to the proposed model.

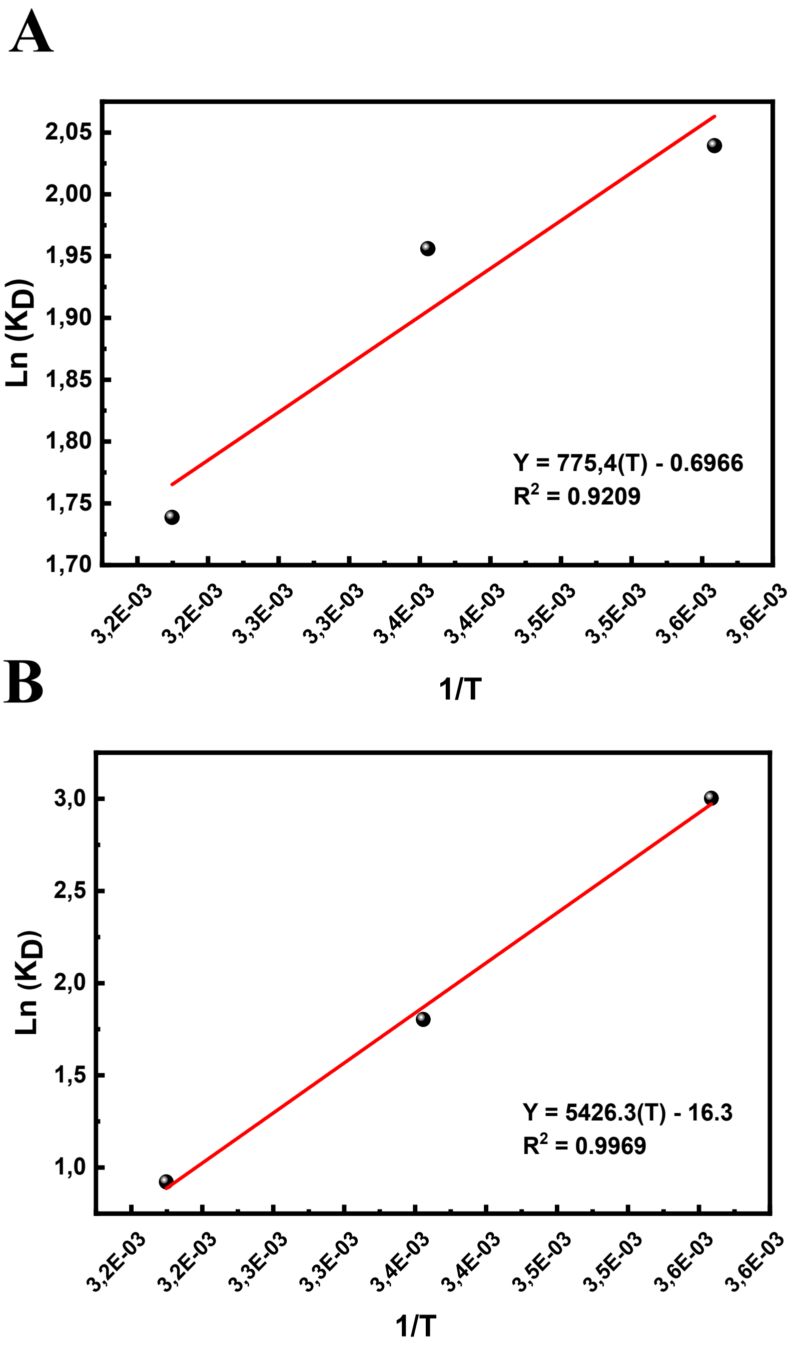

Figure S4. Van't Hoff plots to determine different thermodynamic parameters in the removal of dyes (A) Alizarin Violet and (B) Methylene Blue.

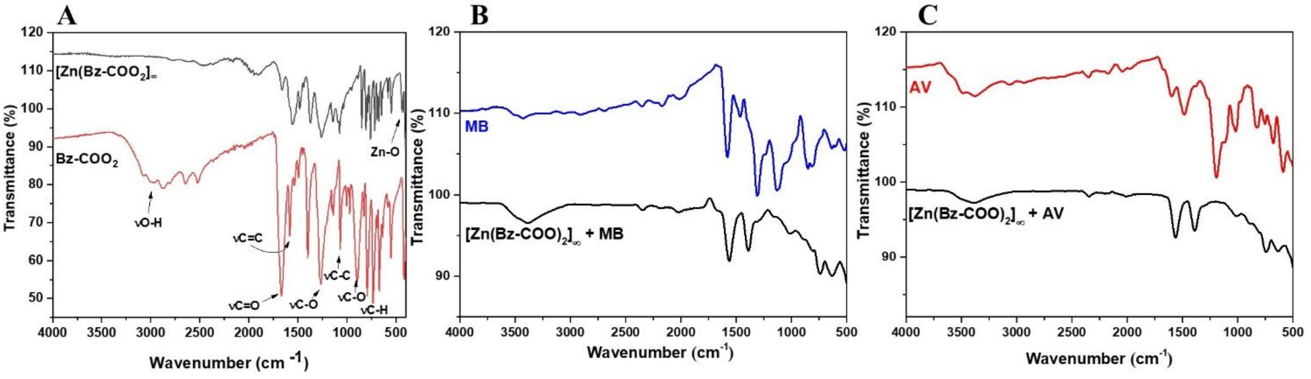

Figure S5. FT-IR spectra of **(A)** pure [Zn(Bz-COO)$_2$]; **(B)** [Zn(Bz-COO)$_2$] after adsorption of methylene blue (MB); and **(C)** [Zn(Bz-COO)$_2$] after adsorption of alizarin violet (AV), highlighting the spectral changes associated with the adsorption process.

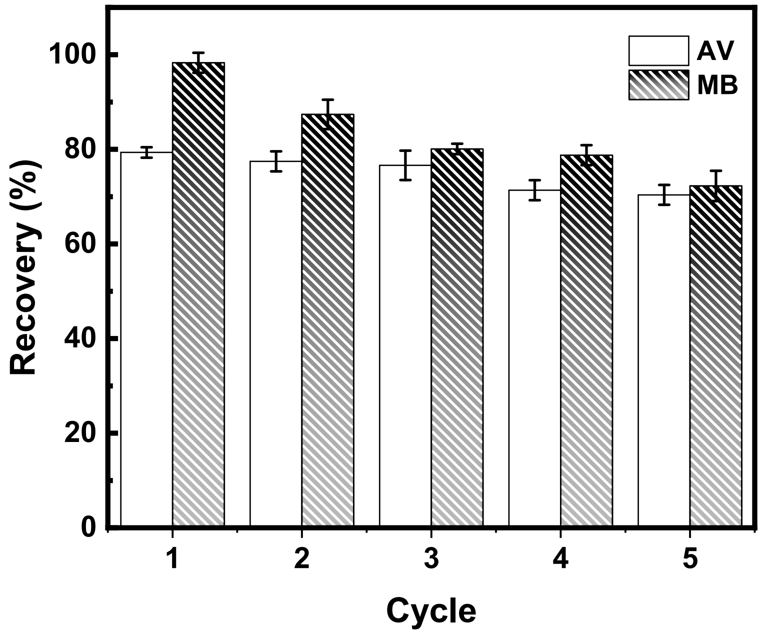

Figure S6. Reuse of Zn(Bz-COO)$_2$]$_\infty$ for adsorption of Alizarin Violet (AV) and Methylene Blue (MB).
